# Single-Cell RNA-Sequencing Reveals the Cellular and Genetic Heterogeneity of Skin Scar to Verify the Therapeutic Effects and Mechanism of Action of Dispel-Scar Ointment in Hypertrophic Scar Inhibition

**DOI:** 10.1155/2022/7331164

**Published:** 2022-06-08

**Authors:** Zhaoyi Li, Libo Yin, Yuanyuan Li, Yi Cao, Haifeng Zeng

**Affiliations:** ^1^The First Clinical College of Zhejiang Chinese Medical University, Hangzhou 310053, Zhejiang, China; ^2^The First Affiliated Hospital of Zhejiang Chinese Medical University, Hangzhou 310006, Zhejiang, China

## Abstract

Hypertrophic scarring (HS), caused by excessive fibrosis of injured skin, imposes a psychological burden and creates a source of distress that impairs the quality of life of affected individuals. However, the gold standard for HS treatment has not yet been determined due to the complicated and difficult nature of the routines and procedures involved. Previous studies have indicated that the topical application of certain active components found in traditional Chinese medicines shows potential as a therapeutic alternative for scars. Here, single-cell RNA-sequencing was performed to determine cellular heterogeneity and identify marker genes and mechanisms associated with HS. It was found that fibroblasts comprise the largest proportion of HS cell types. The marker genes that were highly expressed in fibroblasts were extracellular matrix (ECM)-related, whereas ECM-receptor interactions and the transforming growth factor (TGF)-*β* signalling pathway were also found to be active. Ultra-high-performance liquid chromatography-quadrupole time-of-flight mass spectrometry, which was applied to identify the molecular compounds of Dispel-Scar Ointment (DSO), revealed 74 effective chemical components belonging to 14 types of constituents, such as flavonoids, tanshinones, salvianolic acids, glycosides, and phthalides. Furthermore, *in vivo* studies using rat scar models showed that the topical application of *Salvia miltiorrhiza*, *Ligusticum chuanxiong*, peach kernel, safflower, and motherwort exerted beneficial effects on fibroblasts. DSO promoted scar maturation and reduced scar areas, its efficacy being similar to that of topically applied silicone. Functional studies using immunofluorescence staining, western blotting, and quantitative real-time polymerase chain reaction demonstrated that DSO may target the TGF-*β*/Smad pathway to inhibit collagen synthesis and promote ECM remodelling. However, further *in vitro* mechanistic research and single-drug prescription studies may be required to identify the specific effective compound or active ingredient of DSO, which would provide more substantial evidence regarding the potential therapeutic value of traditional herbs in HS.

## 1. Introduction

Scarring, a natural response of the body, restores skin integrity following injury. Abnormal scarring is a complication of aberrant wound healing caused by excessive proliferation of fibrous tissue. Abnormal scars are divided into three types: hypertrophic, keloid, and atrophic [1]. The wound healing process occurs in three stages: inflammation, proliferation, and remodelling. During proliferation and remodelling, certain fibroblasts that combine with macrophages differentiate into myofibroblasts and synthesise collagen, which, together with other fibroblasts, forms the extracellular matrix (ECM). Excess ECM leads to the degradation of type III collagen, which is remodelled into mature type I collagen. Therefore, scars are characterised by excessive collagen content and abnormal ECM deposition caused by fibroblast proliferation [2]. Effective treatment methods for this condition include surgery, laser therapy, radiation therapy, pressure therapy, cryotherapy, and topical use of silicones, steroids, and injections of botulinum toxin type A [3], which prevent the formation of hypertrophic scars by inhibiting fibroblast activity and suppressing excessive ECM accumulation [4].

Dispel-Scar Ointment (DSO), derived from the Taohong Siwu decoction, is composed of 30 g·*S. miltiorrhiza*, 15 g·*L. chuanxiong*, 30 g peach kernel, 30 g safflower, and 15 g motherwort. Previous studies of ours have shown that DSO is effective against hypertrophic scarring (HS), both *in vivo* and *in vitro* [5, 6]. The chemical constituents of DSO remain ill-defined because of its complexity. In recent years, ultra-high-performance liquid chromatography-quadrupole time-of-flight mass spectrometry (UPLC-Q-TOF-MS) has helped identify various chemical components of traditional Chinese medicine formulae with high sensitivity and resolution. Compounds can be effectively separated using UPLC and then characterised by MS to verify their quality and pharmacological activity for future *in vitro* and *in vivo* studies [7–10]. Therefore, we performed a UPLC-Q-TOF-MS-based metabolomics analysis of DSO to determine its chemical composition, which led to the identification and exploration of a total of 74 compounds, including tanshinone II b, hydroxysafflor yellow A, leonurine, stachydrine, quercetin, and senkyunolide.

Single-cell RNA-sequencing (scRNA-seq) provides opportunities to explore the cellular heterogeneity of skin [11]. ScRNA-seq indicated that fibroblasts in normal human dermis may be divided into multiple subgroups [12]. ScRNA-seq was performed to analyse keloids and revealed that the percentage of a fibroblast subpopulation expressing mesenchymal cell markers, such as POSTN, COMP, COL11A1, COL12A1, and COL5A2, was substantially increased. Further functional research suggested that mesenchymal cells may induce the overexpression of collagen in keloid scars via POSTN. Moreover, compared with that in normal scars, the interaction between transforming growth factor (TGF)-*β* and TGF-*β* receptors in keloids was found to be the most significant, thereby providing a potential target for scar treatment [13].

Therefore, we performed a scRNA-seq analysis of HS tissues to explore marker genes as well as signalling pathways related to hypertrophic scar inhibition, which can affect as the direction of further functional studies. In order to provide an experimental basis for the clinical application of DSO in HS therapy, we performed immunofluorescence staining, western blotting, and quantitative real-time polymerase chain reaction to help us better understand the mechanism of action of Chinese medicines intended for external use in skin diseases.

## 2. Material and Methods

### 2.1. Sample Preparation and Tissue Dissociation

Sprague-Dawley rats (weighing approximately 300 g) aged 6–8 weeks were purchased from Zhejiang Chinese Medical University for the construction of the animal models. All procedures were approved by the Institutional Animal Care and Use Committee of Zhejiang Chinese Medical University (20200914-01). Rats were housed in a standard laboratory under controlled conditions with a room temperature of 22°C (±1°C), a humidity level of 65–70%, and a 12-h light-dark cycle along with free access to food and water.

Following anaesthetisation using intraperitoneally injected pentobarbital (50 mg/kg), rat tail skins were disinfected with iodophor. Using scalpels and iris scissors, a 9 × 9 mm square excisional wound was generated on the tail, and the panniculus carnosus was removed. After haemostasis, the wounds were covered with sterile dry gauze. Twenty-one days were required to fully re-epithelialise and harvest mature hypertrophic scars [14].

The skin tissues were washed twice in Hank's Balanced Salt Solution (HBSS), cut into 5 mm diameter pieces, and immersed in 8 mL EDTA in a 37°C water bath for 40 min. The soaked tissues were resuspended, washed twice in HBSS, and transferred to a 2 mL centrifuge tube. Tissues were quickly cut into 1–3 mm diameter pieces on ice and washed 5 times in HBSS. After the supernatant was removed, a mixed enzyme solution was added. Next, the tissues were digested in a 37°C water bath at 100 rpm for 20–25 min and slowly rotated 5–10 times every 8 min. The resulting cell suspension was filtered through a 30 *μ*m cell strainer and centrifuged at 30 × *g* for 5 min at 12°C. After removing the supernatant, the pellet was washed twice with HBSS, and 5% FBS was added until a cell density of 700–1200 cells/*μ*L was reached. The preparation was then stored on ice for subsequent experiments.

### 2.2. Single-Cell cDNA and Library Preparation

**Figure 1:**
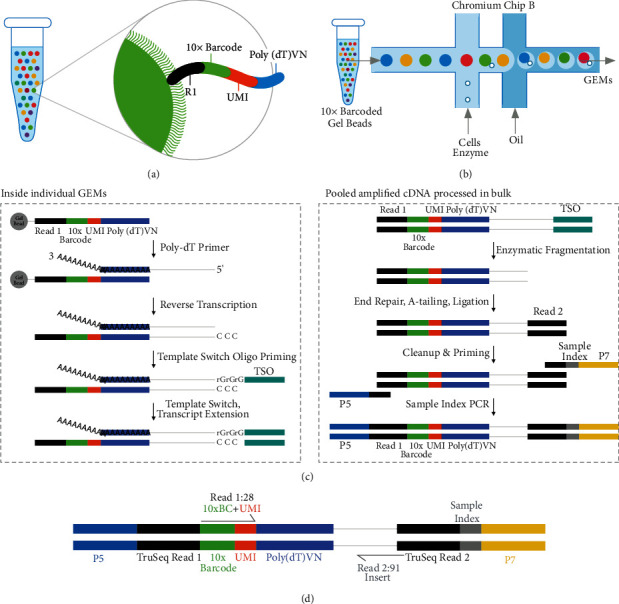
The single-cell RNA-sequencing (scRNA-seq) procedure. (a) GEM structure. (b) Double-cross microfluidic system. (c) Library preparation process. (d) Structure of the library.

Single-cell cDNA library preparation and 3′-end scRNA-seq were performed by Lianchuan (Hangzhou, China) as follows: (i) Chromium™ Single Cell 3′ Solution is a microfluidic platform based on GemCode technology. Gel beads with barcodes, primers, and single cells were wrapped in oil droplets to form gel beads in emulsion (GEM) to obtain a mixture containing barcoded gel beads, cells, and reaction reagents wrapped in oil droplets. The primer composition comprised a full-length Illumina TruSeq Read 1 sequencing primer, 16 bp 10X barcode sequence (for cell distinction), a 12 bp unique molecular identifier (UMI; to distinguish different transcripts of uniform cells and remove polymerase chain reaction (PCR) duplications), and a 30 bp poly(dT) reverse transcription primer (Figure 1(a)). The prepared cell suspension, 10X barcode magnetic gel beads, and oil droplets were added to different channels of Chromium Chip B (eight channels in total), and GEM was formed via the microfluidic “double-cross” system. To obtain a single-cell reaction system, the concentration of the cell suspension was controlled at 700–1200 cells/*μ*L, as a result of which 90–99% of the GEM did not contain cells, whereas a majority of the remaining GEM contained one cell (Figure 1(b)); (ii) cDNA formation and amplification: gel beads of the GEM were dissolved, mRNA was released after cell lysis, and barcoded cDNA for sequencing was produced by reverse transcription. After the liquid oil layer was destroyed, the cDNA was recovered, purified, and amplified to generate a sufficient quantity for library preparation; and (iii) library preparation (Figure 1(c)): after the cDNA was digested into fragments of approximately 200–300 bp, the ends were filled in and an adenine (A) base was added to connect the linker. The kit (LC-Bio Technology Co., Ltd., Hangzhou, China) was used to screen and recycle target size fragments and performed library quality inspection and quantification. Finally, PCR amplification was performed to obtain the DNA library.

### 2.3. 3′-End scRNA-Seq

The paired-end sequencing mode of the Illumina sequencing platform was used for high-throughput sequencing of the DNA library. The constructed library structures from left to right (5′–3′) were (Figure 1(d)) as follows: Illumina P5 adapter, P5 sequencing primer (black Read 1), 16 bp barcode, 12 bp UMI, ploy(dT) VN sequence, inserted cDNA fragment, P7 sequencing primer (black Read 2), sample index (i7 index read), and Illumina P7 adapter. The available sequence length of Read 1 was 28 bp (16 bp barcode + 12 bp UMI), which was used to distinguish between cells and each different transcript (mRNA) of each cell, and Read 2, with a 91 bp sequence length, was used for comparison to the reference genome to determine genetic information.

### 2.4. Single-Cell RNA-Sequence Data Processing

The 10x Genomics official analysis software Cell Ranger (https://support.10xgenomics.com/single-cell-gene-expression/software/overview/welcome) was used to filter, compare, quantify, identify, and recover cells from the original data and obtain the final gene expression matrix of each cell. Subsequently, Seurat (https://satijalab.org/seurat/) was used for further cell filtration, standardisation, cell subgroup classification, analysis of differentially expressed genes in each subgroup, and marker gene screening.

### 2.5. Cell Clusters

The cell clustering process included the following steps: first, low-quality cells were removed using the Seurat R package, version 3.0.1. The LogNormalization method of the “Normalization” function of the Seurat software was then used to normalise expression levels. Next, the normalised expression values were used for principal component analysis. The first 10 principal components were selected for subsequent clustering and grouping analyses. Based on the graph, Seurat software clustering algorithms were used to cluster and group cells by clustering groups exhibiting relationships between cells and optimising the weight value of the distance of the clustering relationship between cells. Finally, cell clusters were identified through a clustering algorithm optimised via the shared nearest neighbour module, and clusters were determined by optimising the modular function. Based on the above results, the nonlinear dimensionality reduction method of t-distributed stochastic neighbour embedding (tSNE) was applied to visualise the results of single-cell subgroup classification.

### 2.6. Fibroblast Reclustering and Comparison of Fibroblasts to Other Cells

The clustering and dimensionality reduction analysis algorithm, tSNE, and the differential analysis algorithm, bimod, were used to identify feasible interactions between and within fibroblasts and other dermal cell populations. The main parameters were as follows: (i) difference screening condition, *p* < 0.01; (ii) difference filter condition log2fc values ≥ 0.26; and (iii) the expression ratio of the differentially screened genes in at least one group was 0.1.

### 2.7. Differentially Expressed Gene Analysis

Seurat was used to determine the genes that were differentially expressed in different cell populations via the bimod likelihood ratio statistical test, as well as to screen upregulated genes. The screening conditions for upregulated genes were as follows: (i) the gene is expressed in >10% cells of the target or control subgroup; (ii) *p* ≤ 0.01; and (iii) log2 FC ≥ 0.26, indicating that the gene upregulation fold was ≥2^0.26.

### 2.8. Gene Ontology (GO) and KEGG Pathway Enrichment Analysis

GO (https://www.geneontology.org/) contains three ontologies that describe the molecular functions of genes, cell locations, and the biological processes involved. GO functional significance enrichment analysis was performed as follows: first, all significantly differentially expressed genes were mapped to each term in the GO database to calculate the gene amount in each term. A hypergeometric test was then performed to determine the significantly enriched GO entries. The results included a histogram and a scatter plot of the enrichment of differentially expressed genes in GO. KEGG (https://www.genome.jp/kegg) is the main public pathway database. Pathway enrichment analysis was performed using the KEGG pathway as the unit, and the same method described above was used to obtain a scatter plot of the enrichment of differential genes in KEGG.

### 2.9. UPLC-Q-TOF/MS Analysis of Bioactive Compounds in DSO

Chinese herbal medicinal components of DSO were provided by the Zhejiang Provincial Hospital of Traditional Chinese Medicine (Zhejiang, China) and identified by the School of Pharmacy, Zhejiang Chinese Medical University. Five herbs were mixed (30 g·*S. miltiorrhiza*, 15 g·*L. chuanxiong*, 30 g peach kernel, 30 g safflower, and 15 g motherwort) in a ratio of 2 : 1 : 2 : 2 : 1 and soaked in five times the amount of 60% ethanol. After approximately 24 h, it was filtered, and the ethanol was recovered and concentrated into a thick paste of 50 mL. It was then dissolved in methanol to obtain stock solutions with appropriate concentrations (1,000–5,000 ng/mL) and centrifuged at 12,000 × *g* for 10 min (Universal 320R; Hettich, Germany). Lastly, the supernatant was filtered using a 0.22 *µ*m syringe filter and refrigerated at 4°C.

Samples of DSO extract were separated into mobile phases consisting of solvents A (aqueous acid water) and B (acetonitrile) using a Waters CORTECS UPLC T3 column (2.1 mm × 100 mm, 1.6 *μ*m; Waters Corp., Milford, MA, USA). An eluting gradient was selected as follows: 0–2 min, 5% B; 2–32 min, 5–100% B; 32–33 min, 100% B; 33.5 min, 5% B; 33.5–35 min, 5% B; the flow rate was 0.3 mL/min, and the sample was injected at a volume of 2 *μ*L for analysis.

UPLC-Q-TOF/MS with an ESI ion source (SYNAPT G2-Si; Waters Corp.) was used to perform TOF/MS data acquisition in positive and negative ion modes and in a centroid mode over the m/z range of 50–1200 Da with a scan rate of 0.2 s. ESI source parameters were set as follows: source temperature of 120°C; desolvation gas temperature of 500°C; cone gas (N_2_) and desolvation gas (N_2_) flow rates of 50 L/h and 1000 L/h in positive ion mode, and 50 L/h in negative ion mode and 800 L/h, respectively; and capillary voltage and cone voltage of 3.0 kV and 40 kV in the positive ion mode, and of 2.5 kV and 40 kV in the negative ion mode, respectively. Collision energy was 6 V for low collision energies and 15–45 V for high collision energies. Sodium formate was used for mass spectrometer calibration, and leucine enkephalin (Sigma-Aldrich, St. Louis, MO, USA; 200 ng/mL; positive ion mode m/z 556.2771, negative ion mode m/z 554.2615) was used for real-time mass calibration.

Raw data were collected using MassLynx V4.1 software and processed using Waters UNIFI software (Waters Corp., Milford, MA, USA) for peak picking. The Traditional Chinese Medicine Systems Pharmacology Database and Analysis Platform (TCMP) database was matched on the Unifi platform to obtain the analysis results of mass spectrometry data initially. The list of possible compounds given by the software was manually identified through the compound cracking rules and related compound mass spectrometry data. In the processing method, the mass error was set to 10 mDa, the error of the retention time was 0.1 min, and the sample threshold intensity was 500 counts.

### 2.10. Study Groups and Topical Medication Interventions

According to traditional Chinese medicine, postsurgery HS is characterised by blood stasis. Its aetiology and pathogenesis are considered as disorders of qi and blood coagulation, respectively. DSO used in this project was derived from an ancient recipe termed the Taohong Siwu decoction. It is composed of Danshen, Chuanxiong, peach kernel, safflower, and motherwort and regulates qi, activates blood, and eliminates stasis.

A total of 25 Sprague-Dawley rats were randomly divided into five groups with five rats in each group as follows: (i) the normal skin (NS) group: no measurement was performed; (ii) the HS model group: only tail scar models were performed; (iii) the silicone group: only silicone ointment (Strataderm Gel; Stratpharma AG, Switzerland) was applied to the tail scars; (iv) the DSO group: only DSO was applied to the tail scars; and (v) DSO + silicone group: DSO and silicone ointment were simultaneously applied to tail scars.

Scar models were created on the tails of rats according to a previously described method [14]. Scar skin was fully re-epithelialised after 21 d. These were then treated with the abovementioned topical medication twice daily for 28 d before harvesting mature hypertrophic scars and normal skin for further research.

### 2.11. Immunofluorescence Staining

The method was as described in [13]. Double immunofluorescence staining of TGF-*β*1, Smad4, Col1a1, and MMP9 was used to evaluate ECM remodelling, deposition of collagen protein, and HS formation. Antibodies against mouse anti-TGF-*β*1(BF8012; Affinity, San Francisco, CA, USA; 1 : 200), rabbit anti-Smad4 (ET1604-12; HUABIO, Woburn, MA, USA; 1 : 200), rabbit anti-MMP9 (ET1704-69; HUABIO; 1 : 200), and mouse anti-Col1a1 (67288-1-Ig; Proteintech, Rosemont, IL, USA; 1 : 200) were incubated overnight at 4°C. After washing thrice with phosphate-buffered saline, goat anti-rabbit IgG-FITC antibody (FITC; 1 : 200; HA1004; HUABIO) or goat anti-mouse IgG H&L (TRITC) antibody (1 : 200; HA1004; HUABIO) was added at 22°C for 1 h. Finally, the samples were stained with 4′,6-diamidino-2-phenylindole (DAPI; Beyotime Biotechnology, Inc., Shanghai, China). Images were obtained using a Nikon A1 confocal laser scanning microscope. All measurements described above were performed with Image-Pro Plus 6.0.

### 2.12. Quantitative Real-Time Polymerase Chain Reaction (RT-qPCR)

The method was as described in [15]. Total RNA was extracted from normal skin and HS tissues of rat tails using TriPure Reagent (BIOFIT, Chengdu, China). Next, cDNA was synthesised from RNA using the Goldenstar RT6 cDNA Synthesis Kit Ver 2 (Tsingke Biotech Co., Beijing, China). RT-qPCR was performed in a 2xT5 Fast qPCR Mix (SYBR Green I; Tsingke Biotech Co.). The mRNA expression levels were normalised to GAPDH expression levels. All data were calculated and quantified using the 2−ΔΔCt method.

The following primers were used:

TGF-*β*1-F: CTGCTGACCCCCACTGATAC

TGF-*β*1-R: GTGAGCACTGAAGCGAAAGC

Smad4-F: TGCATTCCAGCCTCCCATTT

Smad4-R: CCCAAGCAAAAGCGATCTCC

Smad3-F: ATCCGCATGAGCTTCGTCAA

Smad3-R: CCCCAACCCGATCCCTTTAC

Col1-F: GAGACAGGCGAACAAGGTGA

Col1-R: GGGAGACCGTTGAGTCCATC

MMP9-F: TCGGATGGTTATCGCTGGTG

MMP9-R: AAGACGCACATCTCTCCTGC

GAPDH-F: GAAGGTCGGTGTGAACGGAT

GAPDH-R: CCCATTTGATGTTAGCGGGAT

### 2.13. Western Blot

The method was as described in [5]. Skin tissue proteins were isolated using RIPA lysis buffer (25 *μ*L/100 mg). The supernatant was collected following centrifugation at 12,000 × *g* for 15 min at 4°C. A BCA assay was used to assess protein concentrations. An approximately 50 *μ*g protein sample was run on a 10% SDS-PAGE electrophoresis gel, transferred to a PVDF membrane, and blocked at 22°C for 1.5 h with TBST buffer containing 5% dried skimmed milk powder. The membranes were incubated with the following primary antibodies overnight at 4 °C: TGF-*β*1 (1 : 1000; BF8012; Affinity), Smad3 (1 : 1000; ET1604-12; HUABIO), Smad4 (1 : 1000; ET1604-12; HUABIO), Col1a1 (1 : 5000; 67288-1-Ig; Proteintech), MMP2 (1 : 1000; ab92536; Abcam, Cambridge, UK), and MMP9 (ET1704-69; HUABIO). Membranes were subsequently incubated with goat anti-mouse IgG secondary antibody (1 : 5000) or goat anti-rabbit IgG secondary antibody (1 : 5000) at 37°C for 2 h. Antibodies against *β*-actin (1 : 1000; DW130656; Dawen Biotechnology, Zhejiang, China) were used as internal controls. Protein expression was visualised using an electrochemiluminescence microplate reader (FUDE Biological Technology Co., Hangzhou, China).

### 2.14. Statistical Analysis

All data were analysed using GraphPad Prism software, version 8.3, and expressed as the mean ± standard deviation. Student's unpaired *t*-tests were performed to determine the differences between two treatment groups, whereas differences between two or more experimental groups were determined using one-way analysis of variance. All experiments were replicated at least thrice. Statistical significance was set at *P* < 0.05.

## 3. Results

### 3.1. Cellular Heterogeneity of HS Tissues in Transcriptomic Data Generation

**Figure 2:**
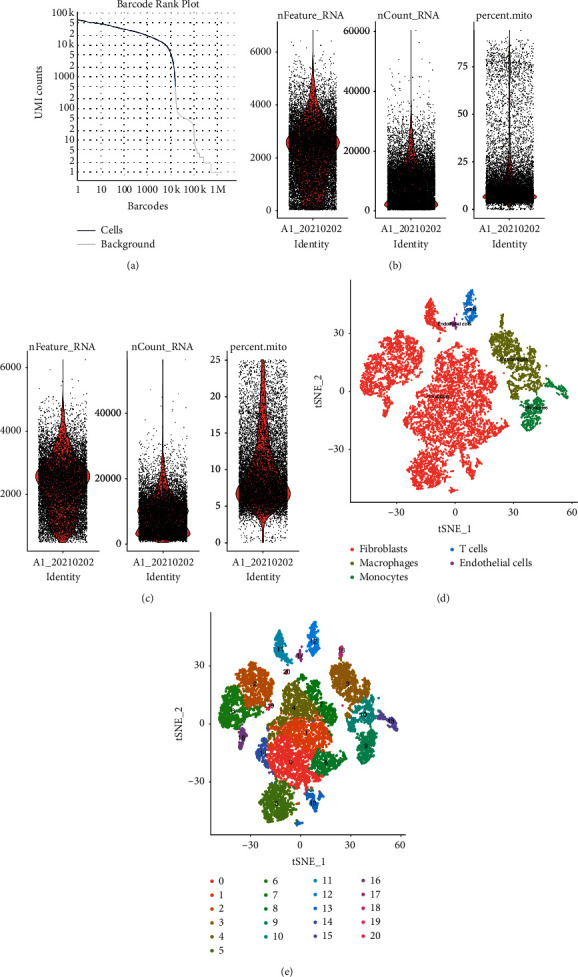
(a) Valid cell identification chart. The abscissa is the barcode sequence number, and the ordinate is the unique molecular identifier number. The barcode corresponding to the blue line refers to the effective cell. (b)–(c) The distribution of basic information of each sample cell before and after filtration ((b) before and (c) after). (d)–(e) Single-cell subpopulation classification tSNE diagram. Each dot represents a cell ((d) different colours indicate cells of different samples, and (e) different colours indicate a different subgroup of cells).

Cell Ranger, the official analytical software of 10x Genomics, was used to analyse the quality statistics of the original data and to compare reference genomes. Following quality control, a whole-transcriptome database of 15,877 cells and 20,915 genes were detected (Figure 2(a)). After poor-quality cells were filtered out using the Seurat R package, version 3.0.1 (Figures 2(b) and 2(c)), the remaining 11,879 cells were subjected to further analysis via single-cell subpopulation classification. Major cell types, including fibroblasts, macrophages, T cells, endothelial cells, and monocytes, were classified into 20 clusters using the LogNormalization technique of the “Normalization” function of the Seurat software. We identified 14 fibroblast clusters (C0, C1, C2, C4, C5, C6, C7, C8, C11, C13, C14, C16, C19, and C20) and three macrophage clusters (C3, C10, and C18) that accounted for a majority of sequenced cells (Figures 2(d) and 2(e)).

### 3.2. Analysis of Fibroblast Cell Subsets

**Figure 3:**
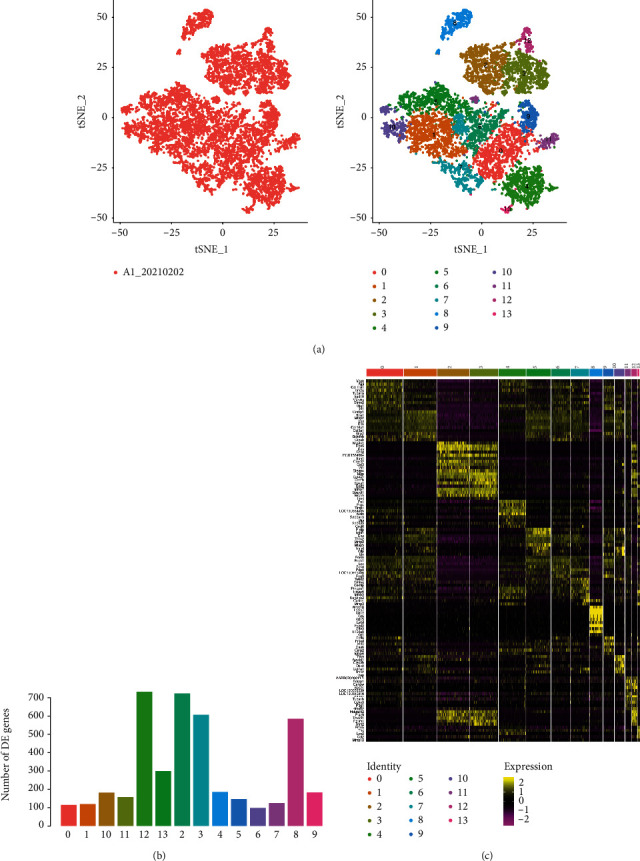
(a) Single-cell subpopulation classification of fibroblasts via the tSNE diagram. Each dot represents a cell. Left image: different colours indicate fibroblasts; right image: different colours indicate different subgroups of fibroblasts. (b) Statistical histogram of the number of upregulated genes in each subgroup. (c) Heat map of marker gene expression in various fibroblast subgroups. Genes with upregulated expression showing the top 10 fold changes in each cluster were selected for heat map construction. Colour indicates the level of the corresponding gene expression in the cell.

During the proliferation and remodelling phase of wound healing, fibroblasts, activated by macrophages, differentiate into myofibroblasts, which synthesise collagen and form the ECM, together with other fibroblasts. Excess ECM undergoes degradation, causing type III collagen to be remodelled into mature type I collagen. Thus, scars are characterised by excessive collagen and abnormal ECM deposition, caused by fibroblast proliferation [2]. In our study, we analysed 8996 fibroblasts and identified differentially expressed genes using scRNA-seq. A total of 13 different fibroblast clusters were found in the scar sample, wherein clusters 0–4 were the top five clusters (Figure 3(a)). However, these clusters could not be differentially identified, and therefore, we were unable to identify the fibroblast type belonging to each cluster. Clusters 2, 3, 8, and 12 expressed the greatest number of upregulated genes (Figure 3(b)).

Clusters 0, 1, 2, 3, 4, 8, and 12 primarily expressed high levels of ECM-related genes (*Des, Dcn, Mgp, Col4a1, Col1a1, Col3a1, Col16a1, Mmp2, Dpt, Sparcl1, Postn, Col3a1, Lum, Vcan, Col11a1, and Tuba1a*) as well as apoptosis-related genes, such as *SOD2* and *Lgals1* (Figure 3(c)). Cluster 8 also exhibited high expression levels of cytokeratin genes derived from skeletal cells (*krt17, krt5, krt15, krt1, and krt14*), which were primarily distributed in epithelial cells and maintained the integrity and continuity of epithelial tissues.

### 3.3. Variation Analysis of Fibroblasts and Other Cell Subsets

**Figure 4:**
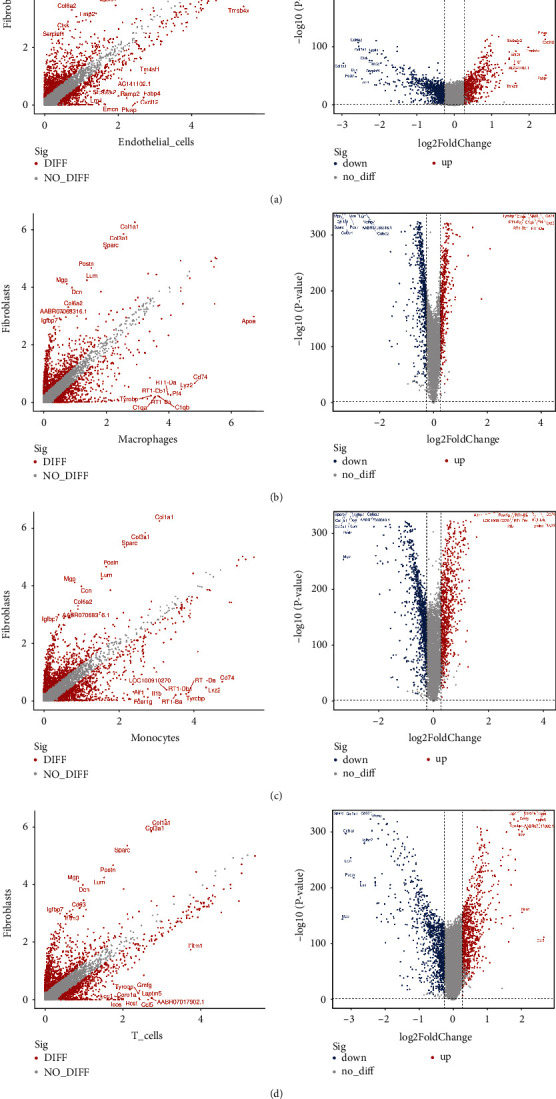
(a)–(d) Differentially expressed genes and volcano maps. Left images: the abscissa and the ordinate represent the treatment and control groups, respectively. The red points represent genes that are significantly differentially expressed between the two groups, and the grey points represent genes that are not significantly differentially expressed between the two groups. The top 10 genes with the largest differential upregulation/downregulation of expression are labelled. Right images: blue indicates genes whose expression is differentially downregulated in the treatment group compared with that in the control group; red indicates genes whose expression is differentially upregulated in the treatment group compared with that in the control group; and grey indicates genes that were not significantly different.

We compared fibroblasts with other cell subsets, such as macrophages, T cells, endothelial cells, and monocytes (Figures 4(a)–4(d)).

As indicated by both scatter and volcano plots, fibroblasts expressed high levels of ECM-related genes, whereas endothelial cells showed high expression levels of *Tmsb4x, Plvap*, and *Emcn*, which regulate cytoskeletal organisation, as well as *Tm4sf1, Fabp4*, and *Ramp2*, which regulate signal transduction. Macrophages exhibited high *Apoe* expression, which is closely associated with *CD74, C1qa*, and *C1qb* that regulate lipoprotein metabolism and immune-related processes. Rano class II histocompatibility antigen genes, *RT1-Db1, RT1-Ba, RT1-Da, CD74,* and *Tyrobp*, which are known to be associated with adaptive immunity, acted as susceptibility genes in monocytes. The levels of potent proinflammatory cytokines (IL1b and Fcer1g) were also increased in monocytes. The actin-binding protein, AIF1, which plays a role in vascular inflammation, was also highly expressed. The IFN-induced antiviral protein, IFITM1, the transmembrane protein, LAPTM5, the cytoskeletal protein, CORO1A, and the transmembrane adapter protein, HCST, were broadly expressed in T cells. Chemoattractant for memory T-helper cells (CCL5), which activates several chemokine receptors, was also increased in T cells. Inducible T-cell costimulator, which is associated with the enhancement of all basic T-cell responses to a foreign antigen, was also highly expressed.

### 3.4. Differentially Expressed Marker Gene (Upregulation) Analysis in Each Cell Subtype

**Figure 5:**
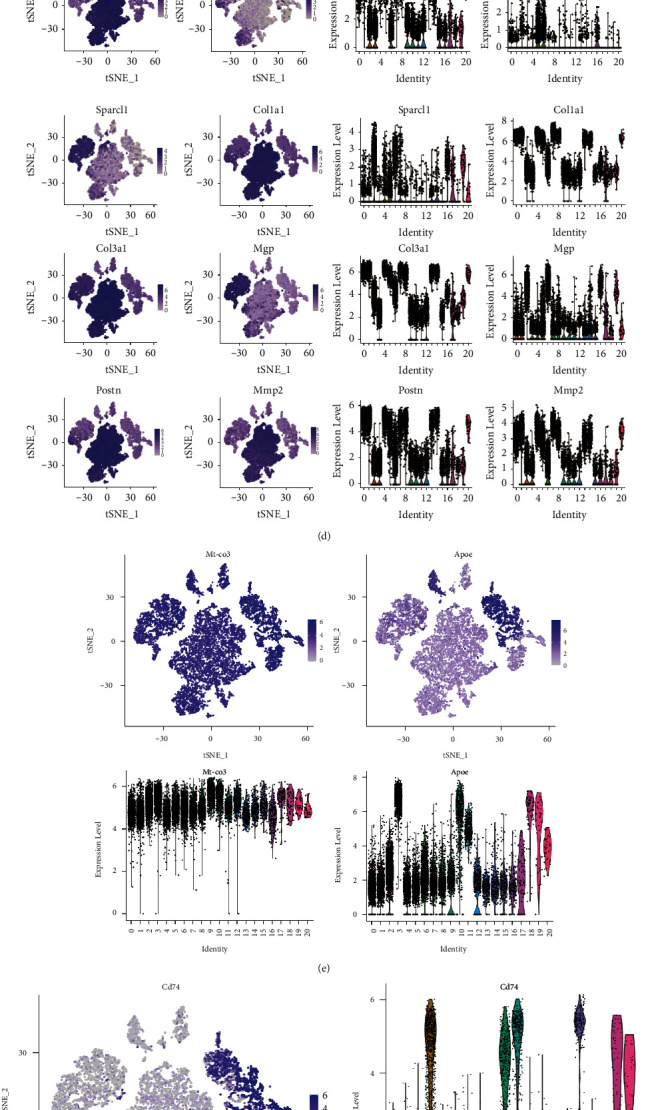
(a) Heat map of marker gene expression in each cell population; colour indicates the expression level of the corresponding gene in the cell. (b) Dot plot map of marker gene expression in each cell population. (c) Ridge map of marker gene expression in each cell population. (d)–(h) Violin graph and tSNE/UMAP graph of marker gene expression in fibroblasts, macrophages, monocytes, T cells, and the endothelial subgroup. Left image: each dot in the figure represents a cell, and the darker the dot, the higher the expression of the marker gene in a specific cell. Right image: the *X*-axis denoted the number of cell subgroups, and the *Y*-axis denotes the expression of the corresponding marker genes in the cells.

The bimod likelihood ratio test was used to analyse differentially expressed genes in each cell cluster, following which the top 10 upregulated genes (log2 FC ≥ 0.26 and *P* ≤ 0.01) in each cluster were selected to determine marked heterogeneity among clusters. Upregulation of TGF-*β*1 increased the level of p-Smad2/3, leading to the upregulation of connexin 43 (Cx43), which affects the phosphorylation of Erk1/2, thereby reducing the synthesis of MMP1, which results in the accumulation of collagen III and the scarring of skin [15]. MMPs are a family of zinc-dependent endopeptidase enzymes that participate in the degradation of various proteins in the ECM. The MMP/TIMP ratio generally determines the degree of ECM protein degradation and tissue remodelling (Figure 5) [16]. We determined that a series of ECM-related genes, such as *Col1a1, Col3a1, Sparcl1, Mgp, Postn, Lum, MMP2,* and *MMP9*, were the most significantly expressed genes in fibroblasts. In addition, various lipoprotein-regulating genes, such as *Apoe* and *Mt-co3*, have been detected in macrophages. Monocytes showed specific expression of CD74, which distinguished them from other clusters. T cells expressed high levels of *Tmsb4x* and ribosomal protein genes, such as *Rplp1, Rplp0,* and *Rps14*. Mitochondrial genes, such as *Mt-co2, Mt-co3,* and *Mt-nd1*, were highly expressed in endothelial cells. In general, our study reconfirmed that the genes previously identified to be related to HS were indeed so and helped explore additional signature gene markers for different cell types.

### 3.5. Gene Set Enrichment Analysis of HS Tissue

Dysregulation of the TGF-*β*1/Smad signalling pathway is an important pathogenic mechanism underlying tissue fibrosis. The TGF-*β* family is involved in cell proliferation, migration, differentiation, deposition, and ECM remodelling [17]. Regulating the TGF-*β*/Smad signalling pathway inhibited the proliferation, invasion, and collagen synthesis of fibroblasts, as well as the formation of hypertrophic scars [18], which findings were consistent with our results.

**Figure 6:**
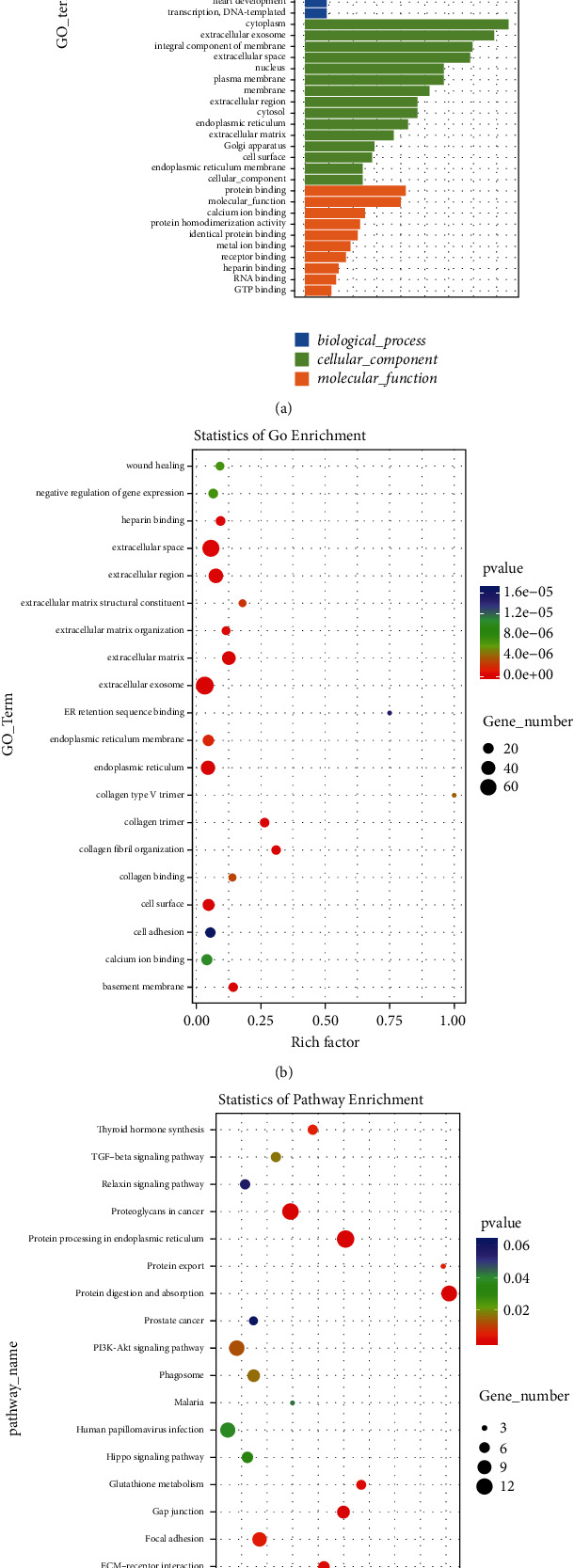
(a). Histogram of differential GO enrichment. The histogram of GO enrichment analysis results reflects the distribution of the number of differential genes in GO terms enriched in biological processes, cellular components, and molecular functions. (b). Scatter plot of GO enrichment of differential genes: the richness factor represents the ratio between the number of differentially expressed genes identified via GO and the total number of genes in GO. The larger the richness factor, the higher the degree of GO enrichment. (c) Scatter plot of KEGG enrichment of differential genes: the richness factor represents the ratio between the number of differentially expressed genes in KEGG and the total number of genes in KEGG. The larger the richness factor, the higher the degree of KEGG enrichment.

To explore cell-subset-specific pathways as well as the properties of genes and gene products in HS tissue, we performed GO enrichment analysis for upregulated genes (Figures 6(a)–6(b)). GO analyses suggested that upregulated genes in most cells were associated with collagen fibril organisation, ECM organisation, wound healing, vasculogenesis, positive regulation of angiogenesis, and proton transmembrane transport. We determined that the cellular components associated with most genes were cytoplasm, extracellular exosomes, integral membrane components, and ECM, whereas the molecular functions associated with these genes were protein binding, calcium ion binding, and protein homodimerisation activity. We also performed KEGG pathway analysis (Figure 6(c)) and found that the TGF-*β* signalling pathway, ECM-receptor interaction, proteoglycans in cancer, and protein processing in endoplasmic reticulum, as well as the PI3K-AKT signalling pathway and the TNF signalling pathway, were activated in most cells, particularly fibroblasts.

Firstly, scRNA-seq analysis revealed that the highly expressed marker genes in fibroblasts were almost ECM-related genes. And disruption of the delicate balance between ECM protein deposition and degradation leads to HS formation [19]. Secondly, gene set enrichment and KEGG pathway analysis via scRNA-seq revealed that the TGF-*β* signalling pathway was activated in most cells, particularly in fibroblasts (Figure 6(c)). As we know, TGF-*β* activation can influence ECM deposition [20]. Although genes of Smads were not explored via scRNA-seq analysis, the Smad family (such as p-Smad2/3) has been proved to be the indispensable part of TGF-*β*1 signal transmission to the nucleus [21]. Besides, evidence increasingly suggests that dysregulation of the TGF-*β*1/Smad pathway is an important pathogenic mechanism underlying HS formation. Therefore, we draw the conclusion that targeting the TGF/Smad pathway may be an effective therapeutic strategy against HS, which needs further experimental verification.

### 3.6. Analysis of DSO via UPLC-Q-TOF-MS and Identification of Its Main Constituents

**Figure 7:**
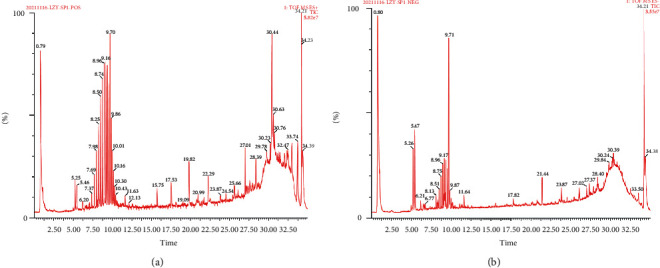
Base peak chromatograms of DSO in positive (a) and negative (b) ion modes.

UPLC-Q-TOF-MS was used to analyse both reference and sample solutions under chromatographic and mass spectrometry conditions to obtain the liquid chromatography-electrospray ionisation-mass spectrometry base peak chromatogram in the positive and negative ion modes (Figure 7).

Data were collected using MassLynx V4.1 software and matched to the TCMP database on the UNIFI platform, and the components and sources of the compounds were inferred and identified using the following: comparison of reference materials, viewing of the MS fragment ion information, literature reviews, and combination of MS fragmentation rules (Table 1). The response of DSO in the positive ion mode was better than that in the negative ion mode. A total of 74 compounds including 12 tanshinones, five chalcone glycosides, two alkaloids, three internal esters, seven glycosides, six phthalides, three phenolic acids, 21 flavonoids, four organic acids, six salvianolic acids, one terpenoid, two organic compounds, one steroid, and one quinone were identified by analysing the components of the positive ion model. In addition, there were 29 compounds in *S. miltiorrhiza*, 14 in *L. chuanxiong*, 15 in peach kernels, 30 in safflower, and five in motherwort. However, not all chemical components have been identified in this experiment, which need to be further analysed and explored.

### 3.7. DSO Inhibits HS via the TGF-*β*/Smad Signalling Pathway

#### 3.7.1. HS in Rat Tails at 7 Weeks after Wounding

**Figure 8:**
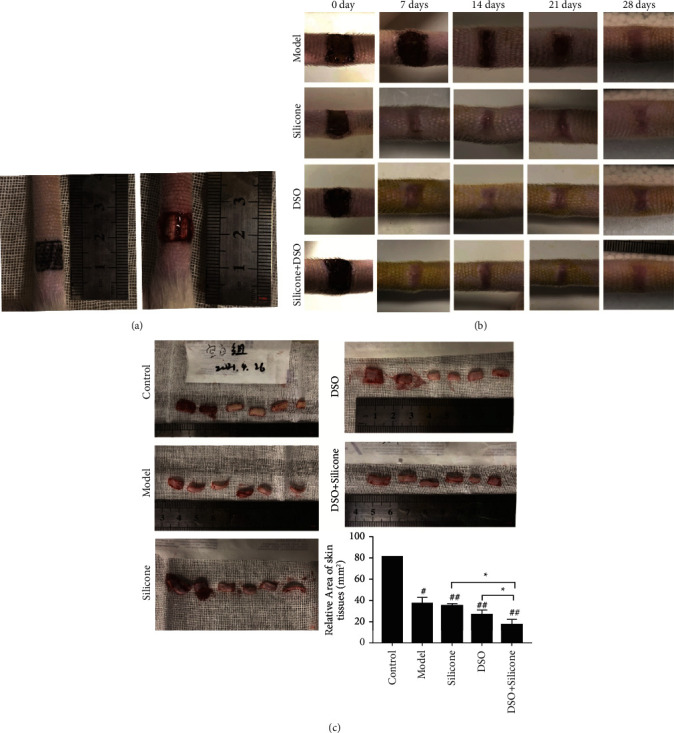
(a) Modelling of HS in rat tails. (b) Representative macroscopic photographic images of HS after being treated with topical medication at 0, 7, 14, 21, and 28 days (scale bars = 5 mm). (c) Mature hypertrophic scars and normal skin tissues of rat tails harvested from the four groups (the HS model group, the silicone group, the DSO group, and DSO + silicone group). Significant differences between different groups or any other groups vs the model group are expressed as ^*∗*^=*P* < 0.05, ^*∗∗*^=*P* < 0.01, and ^*∗∗∗*^=*P* < 0.001, while significant differences between any other group and the control group are expressed as ^#^ = *P* < 0.05, ^##^ = *P* < 0.01, and ^###^ = *P* < 0.001.

The wounding procedure, based on the previously described method, in which 9 × 9 mm square excisional wounds were created on the tail using scalpels and iris scissors together with the removal of the panniculus carnosus, is shown (Figure 8(a)).

Macroscopically, the tail wounds exhibited a moist and granular appearance with minor wound contraction. Fully re-epithelialised scars were treated with topical medication for 28 d, during which we captured photographic images of the different groups on days 7, 14, 21, and 28 (Figure 8(b)). It was apparent that wound healing in the treated group was superior to that in the model group. On day 28, mature hypertrophic scars and normal skin in rat tails were harvested, and the statistics related to the relative scar areas in the four groups were recorded (Figure 8(c)). Differences between the control and model groups were observed. There were significant statistical differences between the silicone alone, DSO alone, and combined DSO and silicone groups (*P* < 0.05), which revealed that the therapeutic effect of the combined application of DSO and silicone was superior to that of silicone or DSO alone.

#### 3.7.2. The Treated Group Regulated ECM Remodelling and Inhibited HS Formation

**Figure 9:**
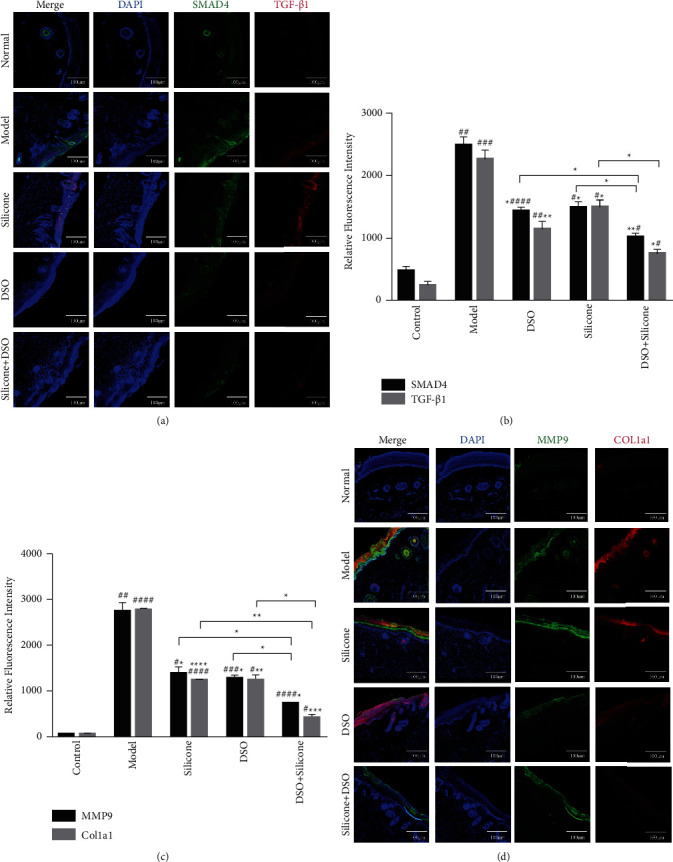
(a) Immunofluorescence staining of TGF-*β*1 and Smad4 in skin tissues. TGF-*β*1 (red), Smad4 (green), and DAPI (blue). Bar = 100 *μ*m. (b) Immunofluorescence staining of Col1a1 and MMP9 in skin tissues. Col1a1 (red), MMP9 (green), and DAPI (blue). Bar = 100 *μ*m. (c) Quantitative analysis of TGF-*β*1-positive and Smad4-positive area density; *n* = 5 for each group. (d) Quantitative analysis of Col1a1- and MMP9-positive area density; *n* = 5 for each group. Significant differences between different groups or any other groups vs the model group are expressed as ^*∗*^=*P* < 0.05, ^*∗∗*^=*P* < 0.01, and ^*∗∗∗*^=*P* < 0.001, while significant differences between any other group and the control group are expressed as ^#^ = *P* < 0.05, ^##^ = *P* < 0.01, and ^###^ = *P* < 0.001.

In order to determine whether the combined application of DSO and silicone regulated ECM remodelling and inhibited the deposition of collagen protein and fibrotic processes, double immunofluorescence staining with TGF-*β*1, Smad4, Col1a1, and MMP9 was used to stain the skin tissues of the five groups (Figure 9). Dermal fibroblasts that stained positive were identified and assessed. The density of fibroblasts showing TGF-*β*1-positive staining in the combination group was lower than that in the other two groups. In addition, Smad4-staining-positive fibroblast density in the combination group was also significantly lower than that in the other two groups. However, no significant difference was observed between the DSO and silicone alone groups. Consequently, these results demonstrated that DSO may inhibit HS formation by regulating the TGF-*β*/Smad signalling pathway.

Concomitantly, the density of Col1a1-staining-positive fibroblasts in the combination group was significantly different compared with that in the DSO and silicone alone groups, indicating that the combined application of DSO and silicone had improved the antifibrosis effect compared with that of the other groups. In addition, the density of MMP9-stained-positive fibroblasts in the combination-treated group was significantly decreased compared with that in the DSO or silicone alone group, suggesting its efficacy for inhibiting ECM deposition. Our results suggested that although DSO exerted a marked dispelling effect on scars, the combined application of DSO and silicone was more effective than the application of DSO or silicone alone.

#### 3.7.3. DSO May Inhibit HS via the TGF-*β*/Smad Signalling Pathway

Evidence increasingly suggests that dysregulation of the TGF-*β*1/Smad signalling pathway is an important pathogenic mechanism underlying tissue fibrosis. The TGF-*β* family is involved in cell proliferation, migration, differentiation, deposition, and ECM remodelling as well as in the regulation of other signalling pathways [17]. In addition, *Astragalus* and *Salvia* extract reportedly inhibited the proliferation, invasion, and collagen synthesis of fibroblasts by regulating the TGF-*β*/Smad signalling pathway to suppress the formation of HS [18]. TGF-*β*1 transmits signals from the activated transmembrane TGF-*β*1 receptor to the nucleus via the Smad family (such as p-Smad2/3) in cells [21]. Studies have indicated that, compared with that in normal fibroblasts, Smad2/3 is overexpressed and highly phosphorylated in scar fibroblasts [22].

**Figure 10:**
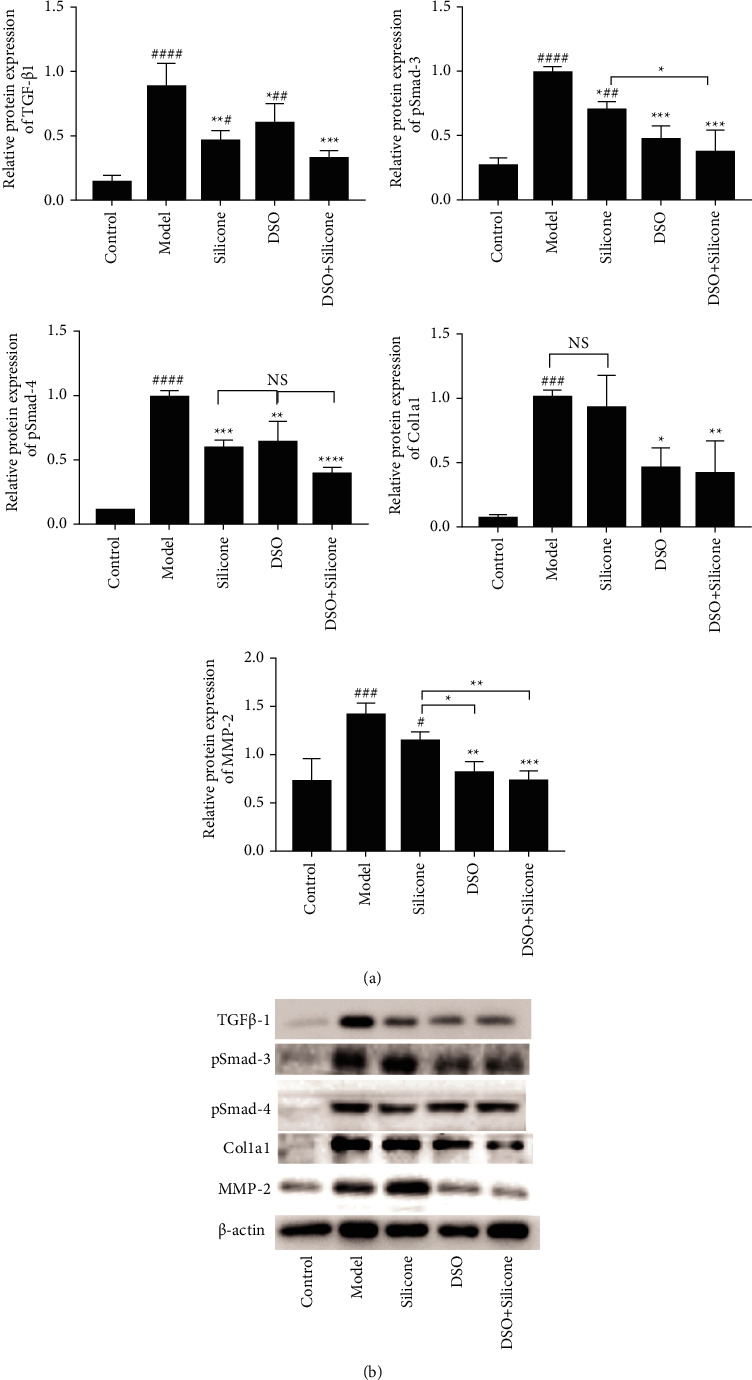
(a) Expression levels of TGF-*β*1, pSmad3, pSmad4, Col1a1, and MMP2 proteins were evaluated via (b) western blotting. *β*-actin levels served as a control (*n* = 3/group). Significant differences among different groups are indicated as ^*∗*^*P* < 0.05, ^*∗∗*^*P* < 0.01, ^*∗∗∗*^*P* < 0.001, vs model group or with any other group; ^#^*P* < 0.05, ^##^*P* < 0.01, and ^###^*P* < 0.01 vs control group.

To evaluate the effect of DSO on Smad3/4 phosphorylation and the subsequent interaction between Col1a1 and MMP2/9, we extracted protein lysates of rat tail skin tissues with or without DSO and conducted immunoprecipitation experiments (Figure 10). The protein levels of TGF-*β*1, pSmad3/4, Col1a1, and MMP2 in the DSO + silicone group were lower than those in the DSO and silicone alone groups. However, a significant difference between the DSO and silicone alone groups was observed only in the expression of MMP2. Interestingly, we also found that pSmad3 expression in the silicone group was significantly different from that in the DSO + silicone group. PCR analysis (Figure 10) of the mRNA expression of TGF-*β*/Smad signalling pathway-related genes (*TGF-β1, Smad3/4*), fibrosis-related genes (*MMP9*), and ECM-related genes (*Col1a1*) confirmed these results.

## 4. Discussion

Recently, scRNA-seq has been widely applied to explore the cellular heterogeneity associated with skin fibrotic diseases and also to define the functionally distinct stromal cell types and marker genes [12, 23], which would enable a better understanding of the pathogenesis of skin fibrosis as well as the identification of potential targets for fibrotic disease treatment. One study used scRNA-seq to demonstrate an increase in the mesenchymal fibroblast subpopulation involved in collagen overexpression in fibrotic skin diseases [13]. Other scRNA-seq analyses have indicated that wound-activated fibroblasts may acquire mesenchymal regenerative capacity to support skin regeneration [24]. The scRNA-seq analysis of rat tail scars conducted in the current study revealed that fibroblasts comprised the largest proportion of HS tissue cell types (Figure 2). Furthermore, comparison between highly expressed genes indicated that the variation between fibroblasts and other cell subsets, such as macrophages, T cells, endothelial cells, and monocytes, was significant (Figures 3–5). However, a subgroup of scar fibroblasts could not be identified, indicating that further research was required.

HS is caused by excessive fibrosis of the injured skin due to damage caused by burns or other insults, which may also involve the joints and mouth. It may impose a psychological burden that acts as a source of distress, impairing the quality of life [25, 26]. Current treatment options for HS include massage therapy, silicone gel treatment, laser therapy, light therapy, radiotherapy, intralesional cryotherapy, and intralesional injection of 5-fluorouracil, interferon, and bleomycin [25, 27]. However, no gold standard for treating HS has been confirmed [28] due to the challenging nature of HS therapy, which involves a complicated and difficult routine [29]. For example, the commonly used method of intralesional injection with corticosteroids leads to after-effects and complications, such as pain and itching [30].

Certain active components of traditional Chinese medicines that are effective in suppressing fibrosis and collagen synthesis show potential as therapeutic agents for scars. Topically applied traditional herbs containing alkaloids and glycosides show considerable wound healing potential [31]. Hong et al. found that *Ligusticum chuanxiong* root extract oil inhibited scar fibroblast proliferation by reducing the levels of TGF-*β*1, MMP1, type I collagen, and type III collagen [32]. Furthermore, experiments have revealed that hydroxy safflower yellow A exerts antifibrotic effects by reducing TGF-*β*1 expression [33]. Fubo et al. reported that a peach kernel coating agent degraded fibrous tissue and reduced scar production by regulating TGF-*β*1 and CD44 [34].

However, molecular components of DSO remain undetermined. UPLC-Q-TOF-MS, regarded as a cogent hyphenated technique for the determination of constituent structures, has been widely applied to analyse the chemical components of traditional Chinese herbs [35, 36]. In the present study, we conducted a UPLC-Q-TOF-MS-based analysis of DSO, which revealed the presence of 14 constituents, including flavonoids, tanshinones, salvianolic acids, glycosides, and phthalides, as well as 74 constituents, including tanshinone II b, hydroxysafflor yellow A, stachydrine, senkyunolide, leonurine, kaempferol, miltirone, quercetin, amarogentin, and lithospermic acid B, among others.

In addition, considerable differences in the healing of wounds and scar areas between the five groups indicated that the therapeutic effects of the combined application of DSO and silicone were superior to those of silicone or DSO alone (Figure 8), which, in turn, may be related to these chemical components. However, the mechanisms underlying the antifibrotic effects of DSO remain unclear.

Our scRNA-seq analysis revealed that the marker genes highly expressed in fibroblasts, such as *Col1a1, Col3a1, Sparcl1, Mgp, Postn, Lum, MMP2*, and *MMP9*, were ECM-related. Moreover, gene set enrichment analysis revealed that biological functions encoded by these genes were related to collagen fibril organisation and ECM organisation, whereas pathway analysis indicated that the ECM-receptor interaction was active in fibroblasts.

The ECM is a granulation tissue composed of procollagen, elastin, proteoglycans, and hyaluronic acid that constitute a repair framework for wound healing and vascular ingrowth. Disruption of the delicate balance between ECM protein deposition and degradation leads to HS formation [19]. When excess ECM is degraded, immature type III collagen is transformed into mature type I collagen [37]. MMPs mediate the breakdown of type I and III collagens [38]. MMP2 and MMP9, in particular, have been found to be active in the ECM remodelling process [39], MMP9 is involved in the degradation of collagen, fibronectin, and elastin, whereas MMP2 plays a key role in degrading denatured collagen and promoting ECM remodelling [40].

The powerful effects exerted by Chinese herbs on ECM remodelling have been demonstrated in multiple studies. Experiments have revealed that high concentrations of hydroxy safflower yellow A inhibit remodelling of the ECM [41]. Other studies have indicated that stachydrine, the active ingredient of motherwort, may regulate the MMP/TIMP system to reduce ECM deposition [42]. Ligustilide inhibits the catabolism of ECM components to maintain ECM homeostasis by downregulating MMPs [43].

**Figure 11:**
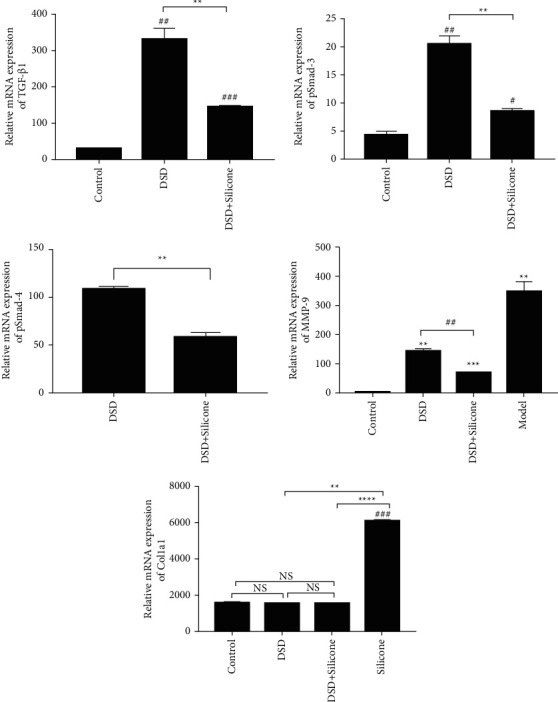
The mRNA expression levels of TGF-*β*/Smad signalling pathway-related genes (*TGF-β1, Smad3/4*), fibrosis-related genes (*MMP9*), and ECM-related genes (*Col1a1*) were detected via RT-qPCR (*n* = 3/group). Significant differences between different groups or any other groups vs the model group are expressed as ^*∗*^=*P* < 0.05, ^*∗∗*^=*P* < 0.01, and ^*∗∗∗*^=*P* < 0.001, while significant differences between any other group and the control group are expressed as ^#^ = *P* < 0.05, ^##^ = *P* < 0.01, and ^###^ = *P* < 0.001.

The results of immunofluorescence staining (Figure 9), western blotting (Figure 10), and RT-qPCR (Figure 11) conducted in the present study showed that DSO markedly suppresses the expression of MMP2, MMP9, and Col1a1. These outcomes indicated that DSO may exert antifibrotic effects in HS primarily through the inhibition of ECM deposition.

Gene set enrichment analysis via scRNA-seq revealed that the TGF-*β* signalling pathway was activated in most cells, particularly in fibroblasts (Figure 6). Chen et al. reported that TGF-*β*1 expression was increased in HS [44]. In response to a specific TGF-*β*, the SMADs downstream of the TGF-*β* type I receptor may be phosphorylated to form a complex with Co-SMAD4, which translocates to the nucleus and regulates the transcription of specific genes [45]. R-SMADs 3 and 4 have been shown to be prominent mediators of autocrine stimulation by TGF-*β* in HS-derived fibroblasts [46], and a decrease in SMAD3 expression led to a reduction in procollagen gene expression and ECM deposition [47]. TGF-*β* activation also leads to excessive ECM deposition [20]. Moreover, TGF-*β*1 may increase MMP2 and MMP9 mRNA and protein levels [48]. In addition, activation of MMP2 may reverse profibrogenic TGF-*β*1 signalling, leading to fibrosis and collagen deposition [49]. Therefore, the TGF-*β*1/Smad pathway appears to play a key role in HS formation by inducing the differentiation of fibroblasts into myofibroblasts [50], inhibiting MMPs, stimulating production, and suppressing ECM degradation [51].

Flavonoids (quercetin and kaempferol) are found in typical topical scar creams, such as Mederma skin care gel (Merz Pharmaceuticals, Frankfurt, Germany) and Contractubex gel (Merz Pharmaceuticals). Interestingly, quercetin, a dietary bioflavonoid, has been shown to inhibit the proliferation, collagen production, and contraction of HS-derived fibroblasts by inhibiting Smad2/3/4 expression [52]. She et al. reported that the inhibitory effects of *S. miltiorrhiza* extract on fibroblasts and collagen synthesis may be mediated via TGF-*β*/Smad signalling that inhibits scar hyperplasia [53]. Notably, sodium tanshinone II A sulfonate attenuated the formation and contracture of HS by reducing the expression of TGF-*β*1 and *α*-SMA [54]. Studies have verified that stachydrine, the active ingredient of motherwort, inhibits tissue fibrosis via the TGF-*β*/Smad pathway and reduces ECM deposition [42].

Immunofluorescence staining (Figure 9), western blotting (Figure 10), and RT-qPCR (Figure 11) conducted by the present study demonstrated that DSO inhibits the protein and mRNA expression of TGF-*β*1 and thereby the phosphorylation of Smad3 and Smad4. Therefore, we hypothesised that suppression of the TGF-*β*/Smad pathway by DSO underlies its inhibitory effects on HS.

In conclusion, the present study demonstrated that DSO inhibits collagen synthesis and promotes ECM remodelling in HS *in vivo*. Thus, these inhibitory effects may be due to the effects exerted by DSO on the TGF-*β*/Smad pathway. However, further mechanical as well as single drug prescription studies *in vitro* are required to identify the specific active ingredient in the DSO formula, which may provide definite evidence for the potential therapeutic use of DSO in HS.

## Figures and Tables

**Table 1:** Compounds identified in DSO via UPLC-Q-TOF-MS.

Identify	Source^a^	Compound Types^b^	Molecular Formula	RT (min)	m/z
Tanshinone II b	1	1	C19H20O3	19.82	297.1489
Tanshinone II A	1	1	C19H18O3	22.29	295.1329
Hydroxysafflor yellow A	4	2	C27H32O16	5.25	613.1773
Stachydrine	5	3	C7H13NO2	0.85	144.1019
Senkyunolide	2	4	C12H16O2	15.75	193.1225
Leonurine	5	3	C14H21N3O5	7.1	312.1553
1,2,5,6-tetrahydrotanshinone I	1	1	C18H16O3	18.2	281.1168
Tanshinone II b	1	1	C19H20O3	29.32	297.1486
Amarogentin	3	5	C20H27NO11	5.47	480.1474
Tanshinone I	1	1	C18H12O3	19.92	277.0854
4-Hydroxy-3-butylphenyl peptide	2	6	C12H14O3	9.24	207.1013
Lithospermic acid B	1	7	C36H30O16	9.71	741.1428
Kaempferol	4	8	C15H10O6	8.34	287.055
Apigetrin	4	8	C21H20O10	5.25	433.113
Tanshinone II b	1	1	C19H20O3	19.99	297.148
3,4-Dihydroxycinnamunic acid	1, 2	9	C9H8O4	9.71	181.0495
Kaempferol-3-O-rutinoside	4	5	C27H30O15	6.54	595.166
Prunasin	3	5	C14H17NO6	6.21	318.0942
Miltirone	1	1	C19H22O2	23.02	283.1692
Salvianolic acid C	1	10	C26H20O10	9.71	493.1131
Salvianolic acid G	1	10	C18H12O7	9.24	341.0652
Apigetrin	4	8	C21H20O10	6.54	433.1126
Salvianolic acid G	1	10	C18H12O7	9.71	341.0664
Cryptomeriol	/	11	C20H28O2	20.24	301.2154
Quercetin	3, 4	8	C15H10O7	7.39	303.0496
Safflower yellow A	4	2	C27H30O15	5.25	595.1663
Z-6,8′,7,3′-dipolymer ligustilide	2	6	C24H28O4	22.5	381.2061
Isotanshinone II A	1	1	C19H18O3	22.47	295.1324
Hydroxysafflor yellow A	4	2	C27H32O16	0.81	613.1773
Salvianolic acid A	1	10	C26H22O10	9.71	495.1278
Alkanoic acid	1	7	C27H22O12	9.71	539.1187
6-Hydroxykaempferol	3、4	8	C15H10O7	7.68	303.0493
Rutin	4、5	8	C27H30O16	7.24	611.1615
Quercetin-3-O-*α*-L-rhamnoside	3, 4	5	C21H20O11	8.34	449.1088
4-Hydroxy-3-butylphenyl peptide	2	6	C12H14O3	9.67	207.1012
Quercetin	3, 4	8	C15H10O7	7.24	303.0497
Rutin	4, 5	8	C27H30O16	7.68	611.161
Prunasin	3	5	C14H17NO6	5.47	296.1126
Kaempferol	4	8	C15H10O6	7.37	287.0542
Neocnidilide	2	6	C12H18O2	17.4	195.1371
Kaempferol	4	8	C15H10O6	8.61	287.055
Methyl tanshinonate	1	10	C20H18O5	18.74	339.1219
Lithospermic acid	1	7	C27H22O12	9.24	561.1004
Hydroxytanshinone II A	1	1	C19H18O4	15.58	311.1276
Quercetin	3, 4	8	C15H10O7	7.93	303.0497
6-Hydroxykaempferol	3, 4	8	C15H10O7	6.77	303.0495
Z-6,8′,7,3′-dipolymer ligustilide	2	6	C24H28O4	22.22	381.2057
Quercetin	3, 4	8	C15H10O7	6.67	303.0497
Miltionone I	1	10	C19H20O4	13.05	313.1441
3,4-Dihydroxycinnamunic acid	1, 2	9	C9H8O4	6.58	181.05
Celery alcohol	4	12	C15H10O5	11.92	271.0598
Hydroxytanshinone II A	1	1	C19H18O4	15.04	311.127
Genkwanin	1, 2	8	C16H12O5	15.27	285.0756
Senkyunolide	2	4	C12H16O2	15.92	193.1222
6-Hydroxykaempferol-3-O-glucoside	3, 4	8	C21H20O12	1.55	465.1031
Protocatechuic aldehyde	1	12	C7H6O3	9.71	139.0398
4-Hydroxy-3-butylphenyl peptide	2	6	C12H14O3	14.38	207.1012
Isoquercetin	3, 4	8	C21H20O12	6.77	465.1028
Hydroxytanshinone II A	1	1	C19H18O4	15.36	311.1274
Safflower yellow A	4	2	C27H30O15	6.85	595.1666
Rutin	4, 5	8	C27H30O16	6.9	611.1612
Safflomin C	4	2	C30H30O14	9.4	615.1712
3,4-Dihydroxycinnamunic acid	1, 2	9	C9H8O4	7.1	181.0492
Danshen neoquinone C	1	14	C16H12O3	19.81	253.0865
Lonicerin	1, 2	8	C15H10O6	7.72	287.0549
Quercetin	3, 4	8	C15H10O7	7.81	303.0497
Apigetrin	4	8	C21H20O10	6.85	433.1127
Hydroxytanshinone II A	1	1	C19H18O4	15.69	311.1275
Senkyunolide	2	4	C12H16O2	12.15	193.1217
Kaempferol-3-O-rutinoside	4	5	C27H30O15	6.36	595.1658
Cryptoacetalide	1	13	C18H22O3	21.32	287.1651
6-Hydroxykaempferol-7-O-glucoside	3、4	5	C21H20O12	7.39	465.1034
Benzoic acid	4	9	C7H6O2	15.75	123.0439
6-Hydroxykaempferol-3-O-glucoside	3, 4	8	C21H20O12	7.68	465.1037

^a^1, 2, 3, 4, and 5 indicate *S. miltiorrhiza*, *L. chuanxiong*, peach kernel, safflower, and motherwort, respectively; ^b^1, 2, 3, 4, 5, 6, 7, 8, 9, 10, 11, 12, 13, and 14 indicate tanshinones, chalcone glycosides, alkaloids, internal esters, glycosides, phthalides, phenolic acids, flavonoids, organic acids, salvianolic acids, terpenoids, organic compounds, steroids, and quinones, respectively.

## Data Availability

The data are available on request to the authors.
